# A Konjac Glucomannan-Based Antibacterial Packaging Film with Humidity-Triggered Release of Cinnamaldehyde

**DOI:** 10.3390/foods15030464

**Published:** 2026-01-29

**Authors:** Yibin Chen, Hao Liu, Kaijun Sun, Qibiao Weng, Ying Yan, Liping Xiao, Ziwei Ye, Chengrong Wen, Jie Pang, Qian Ning

**Affiliations:** 1 College of Food Science, Fujian Agriculture and Forestry University, Fuzhou 350002, China; 2 Fujian Dawuyi Green Food Research and Development Center, Nanping 353000, China; 3 Fujian Provincial Key Laboratory of Eel Breedingand Processing, Fuzhou 350003, China; 4 Jinshan College of Fujian Agriculture and Forestry University, Fuzhou 350002, China

**Keywords:** konjac glucomannan, cinnamaldehyde, relative humidity, controlled release, antibacterial

## Abstract

To meet the challenge of microbial contamination of food, smart packaging materials with active controlled-release functions have become a research hotspot. In this study, a humidity-responsive antimicrobial composite film was constructed by introducing cinnamaldehyde@β-cyclodextrin inclusion complexes (CIN@β-CD ICs) into a konjac glucomannan/polyvinyl alcohol/lithium chloride (KGM/PVA/LiCl) matrix. Characterization results showed that the CIN@β-CD ICs formed a dense structure through hydrogen bonding, which enhanced the thermal stability, mechanical strength (tensile strength: 20.83 MPa) and surface hydrophilicity (water contact angle < 60°) of the film. The film acted as a humidity-triggered release system for CIN, enabling controlled antimicrobial delivery: at high humidity (98% RH), the film rapidly swelled and accelerated the release of CIN, with a cumulative release rate of 87.29% over 7 days, whereas the release slowed significantly at low humidity (43% RH). The antimicrobial activity of the released CIN was strongly influenced by ambient humidity, with the effect enhanced under high humidity conditions. It is noteworthy that the film containing 0.2% ICs exhibited the optimal antimicrobial performance among the formulations studied. This study elucidates a mechanism for humidity-triggered release through multicomponent synergism, which provides a feasible strategy for the design of environmentally friendly, smart packaging materials with high antimicrobial activity.

## 1. Introduction

Food spoilage and the occurrence of foodborne illnesses are frequently attributed to microbial contamination and result in the loss of approximately 30% of food production annually [1]. Conventional food packaging serves as a passive barrier, with its function limited to resisting the intrusion of external microorganisms [2]. To provide more proactive protection, active packaging systems capable of releasing antimicrobial agents have been developed. These systems are engineered to trigger the release of active components in response to specific stimuli, including changes in external environmental conditions (e.g., temperature, humidity, or light) or in the food’s internal microenvironment (e.g., pH) [3,4,5]. Given the strong correlation between high-humidity conditions and concurrent processes of moisture uptake, mold growth, and microbial proliferation in foods, humidity-responsive packaging has attracted considerable research attention [6,7].

The hydrophilicity of packaging materials is fundamental to achieving humidity-responsive functionality [8]. Konjac glucomannan (KGM) is a water-soluble dietary fiber and viscous polysaccharide, valued for its excellent film-forming properties, biocompatibility, and biodegradability in sustainable material applications [9]. Its molecular chains contain abundant hydrophilic hydroxyl groups, which provide ample sites for moisture adsorption and render KGM an ideal humidity-responsive substrate [10]. However, pure KGM films suffer from poor mechanical properties. Forming composite systems with two or more polymers represents an effective strategy to enhance film performance. Polyvinyl alcohol (PVA), a widely used synthetic polymer, also features water solubility, biodegradability, and a hydrophilic chain structure [11]. Blending PVA with KGM promotes the establishment a physically cross-linked hydrogen-bonded architecture, thereby improving the film’s mechanical strength and structural stability [12]. Moreover, conventional humidity-responsive mechanisms primarily depend on changes in polymer chain spacing [13]. Lithium chloride (LiCl) is a highly hygroscopic salt that can efficiently capture ambient water molecules, promote their penetration into the polymer network, increase chain spacing, and accelerate film swelling under high-humidity conditions, as demonstrated in prior studies [14]. Although KGM/PVA/LiCl composite films possess favorable structural strength and humidity responsiveness, they intrinsically lack sufficient antibacterial activity. Consequently, incorporating efficient antibacterial components is essential to advance the functionality and practical utility of this material system.

Cinnamaldehyde (CIN), a natural bioactive compound extracted from cinnamon bark, demonstrates potent broad-spectrum antimicrobial activity against foodborne pathogens [15]. The antibacterial mechanism of CIN is primarily attributed to its interference with the glycerophospholipid biosynthesis pathway in target microorganisms, ultimately compromising cell membrane integrity [16]. However, the high volatility and limited aqueous solubility of CIN restrict its viability for food packaging applications [17]. To counter these limitations, encapsulation strategies including the use of metal–organic frameworks, cyclodextrins, and starch have been explored to improve CIN’s stability and enable its controlled release [7,18]. Notably, β-cyclodextrin (β-CD) is a cyclic oligosaccharide characterized by a lipophilic interior and a hydrophilic outer surface, a structure that facilitates the encapsulation of small hydrophobic molecules [19]. The host-guest interactions between CIN and β-CD prove to be a highly effective strategy, primarily through the development of stable host-guest complexes [20]. For instance, studies have shown that β-CD encapsulated citral and trans-CIN exhibit enhanced thermal stability and prolonged release profiles [21]. Another related study demonstrated the integration of CIN into β-CD’s hydrophobic interior, a process which allowed for the combination of prolonged release characteristics with maintained antibacterial activity [22]. Therefore, β-CD represents a highly suitable delivery vehicle for triggered release of antimicrobial agents.

Based on the above research background, this study aims to construct a high-performance, humidity-responsive antimicrobial film by integrating CIN@β-CD ICs into KGM/PVA/LiCl matrix. Although the components (KGM/PVA, LiCl, CIN@β-CD) have been reported, this study focuses on elucidating their synergistic effects and action mechanisms within this composite system, especially in jointly optimizing humidity-triggered release kinetics and antibacterial performance. To this end, we first synthesized and characterized CIN@β-CD ICs, then systematically investigated their incorporation effects on the microstructure, mechanical properties, and humidity-responsive behavior of the films. Subsequently, the release behavior of CIN under different relative humidity (RH) conditions and its corresponding antimicrobial efficacy were evaluated. This study not only provides a multi-component integration strategy for the development of advanced active packaging materials, but also offers a theoretical basis for the design and optimization of such intelligent packaging through an in-depth analysis of the relationship between release mechanisms and structure-performance.

## 2. Materials and Methods

### 2.1. Materials

Konjac glucomannan (KGM, Mw = 1.96 × 105 Da, purity ≥ 90%) was obtained from Yizhi Konjac Biological Technology Co., Ltd. (Yichang, China). Poly (vinyl alcohol) (PVA, Mw = 31,000–50,000), cinnamaldehyde (CIN, GC ≥ 95%) were acquired from Aladdin Biochemical Technology Co., Ltd. (Shanghai, China). β-cyclodextrin (β-CD), Lithium chloride (LiCl), and other reagents were of analytical grade and supplied by Sinopharm Chemical Reagent Co., Ltd. (Shanghai, China).

### 2.2. Preparation of CIN@β-CD ICs

A modified co-precipitation approach, following the protocol described by Zhang et al., was employed to synthesize the CIN@β-CD ICs [23]. Briefly, complete dissolution of β-CD (5 g) was achieved by stirring in 100 mL distilled water at 60 °C for one hour. Following this, a CIN-ethanol solution (6 mL, 12.5% *w*/*v*) was introduced dropwise into the resulting β-CD solution with continuous stirring over a period of 4 h. The resulting mixture was maintained at 4 °C for 24 h to promote precipitation. The suspension was then centrifuged at 8000 rpm for 30 min to collect the precipitate. Finally, the obtained solid was freeze-dried for 48 h to yield the CIN@β-CD ICs.

### 2.3. Characterization of CIN@β-CD ICs

To determine particle size, an appropriate amount of CIN@β-CD ICs powder was dispersed in an aqueous medium, followed by measurement of the resulting dispersion with a laser diffraction particle size analyzer (Malvern, Mastersizer 3000,Worcestershire, UK).

The absorbance at 280 nm of the collected supernatant was measured with a UV-2600 spectrophotometer (Shimadzu, Kyoto, Japan) after the CIN@β-CD ICs were prepared [22]. The CIN concentration was quantified based on a pre-established calibration curve. The encapsulation efficiency (EE) and loading capacity (LC) were calculated according to the following equations:

(1) $$\mathrm{EE}\;\left(\%\right)=\;\frac{M_{t}-M_{f}}{M_{t}}\;\times \;100$$

(2) $$\mathrm{LC}\;\left(\%\right)=\frac{M_{t}-M_{f}}{M_{n}}\;\times \;100$$

where *M_t_* is weight of encapsulated CIN, *M_f_* represents the weight of free CIN in the supernatant, and *M_n_* is the weight of the inclusion complexes.

### 2.4. Preparation of KGM/PVA/LiCl/ICs Film

The initial step involved dissolving KGM and PVA (3:2) in 100 mL deionized water, which contained 0.05% (*v*/*v*) glycerol as an added plasticizer, and then the mixture was stirred at 80 °C for 2 h. Subsequently, 0.25% LiCl was added and stirring continued for 1 h to ensure complete dissolution, yielding the KGM/PVA/LiCl film-forming solution. Next, varying amounts of CIN@β-CD ICs powder (0.1%, 0.2%, and 0.3% relative to the solution mass) were incorporated into the base solution, followed by stirring at 60 °C for 3 h and ultrasonication for 10 min. The resulting mixture was cast into disposable Petri dishes and allowed to dry in a laboratory environment maintained at 50% RH. The final films were designated as KPL, KPLC-1, KPLC-2, and KPLC-3, corresponding to the increasing content of CIN@β-CD ICs.

### 2.5. Characterization of KGM/PVA/LiCl/ICs Film

#### 2.5.1. Structural Characterization

To comprehensively analyze the composite films, a series of characterization techniques were employed. Morphological examination of both surface and cross-sectional features was performed via scanning electron microscopy (SEM, ZEISS, Sigma 360, Oberkochen, Germany). Chemical structure and functional groups were investigated using Fourier transform infrared (FTIR, Thermo Fisher, Scientific Nicolet iS20, Waltham, MA, USA) spectroscopy in attenuated total reflectance (ATR) mode. Crystalline phases within the samples were identified by X-ray diffraction (XRD, Bruker, D8 Advance, Karlsruhe, Germany). This multi-faceted analytical approach provided essential insights into the films’ microstructure, molecular composition, and crystallinity.

#### 2.5.2. Thermal Stability Analysis

The thermal stability of the films was evaluated using a thermogravimetric analyzer (TGA, Netzsch, STA 409PC, Selb, Germany) at an ambient temperature of 25 °C. The samples were heated in a nitrogen atmosphere over a temperature range of 30 °C to 600 °C, with a heating rate of 10 °C/min until reaching 600 °C.

#### 2.5.3. Mechanical Properties

The mechanical properties of the films, including tensile strength (TS, MPa) and elongation at break (EAB, %), were measured using an electronic tensile testing machine [24]. Prior to testing, the films were conditioned at 50% RH and 25 °C for 48 h. The films were cut into rectangular samples measuring 10 mm × 30 mm and tested at a gauge length of 20 mm with a testing speed of 5 mm/min.

#### 2.5.4. Water Contact Angle (WCA)

The WCA was determined with a video-based goniometer (Dingsheng, JY-82C, Chengde, China) to evaluate surface wettability. Film specimens (2 cm × 2 cm) were positioned on the sample stage, and a 10 μL droplet of distilled water was deposited on the surface using a precision micro-syringe. For statistical reliability, WCA readings from three distinct locations per sample were averaged [25].

#### 2.5.5. Water Vapor Permeability (WVP)

A gravimetric method was employed to determine the WVP. Precisely 3.00 g of anhydrous CaCl_2_ was weighed into individual glass vials. Each vial opening was hermetically sealed with a film specimen and secured tightly. These assemblies were subsequently stored in a humidity-controlled desiccator at 25 °C and 75% RH. Gravimetric data were collected every 24 h over a period of 72 h, and the WVP was then calculated using the following relationship:

(3) $$WVP(g\;mm/m^{2}h\;kPa)=\frac{∆M\times d}{S\times ∆t\times ∆P}$$

where ΔM denotes the cumulative mass gain (g); d is the film thickness (mm); S represents the exposed film area (m^2^), Δt is the interval between measurements (h), and ΔP signifies the water vapor pressure difference (kPa).

#### 2.5.6. Moisture Absorption (MS)

The films’ ability to absorb moisture was quantified using a stepwise drying and rehydration process, in accordance with a reported procedure [26]. Drying of the film samples was carried out at 105 °C for 24 h in an oven to acquire their dry mass. Subsequently, the samples were sealed in glass desiccators maintained at 25 °C and different RH levels of 43%, 75%, and 98%. These specific RH conditions were generated using saturated solutions of K_2_CO_3_, NaCl, and K_2_SO_4_, respectively. Storage in the desiccators continued until the samples achieved an equilibrium mass. The MS was then calculated using Equation (4), enabling a precise evaluation of the moisture-sorption behavior of the films under various ambient conditions.

(4) $$\mathrm{MS}\;(\%)\;=\frac{M_{2}-M_{1}}{M_{1}}\;\times \;100$$

where M_1_ is the constant weight after drying, and M_2_ is the constant weight after moisture absorption.

### 2.6. Humidity-Responsive Release

Under controlled relative humidity conditions, the liberation of CIN from the KPLC films was examined. Saturated salt solutions of K_2_SO_4_, NaCl, and K_2_CO_3_ were used to maintain constant RH levels of 98%, 75%, and 43%, respectively, in sealed desiccators [27]. Film samples (15 mg each) were placed in the respective RH environments for 7 days. At 24 h intervals, one sample was removed and immersed in 95% ethanol, followed by ultrasonication for 30 min to extract the remaining CIN. The absorbance of the filtrate was measured at 280 nm using a UV-Vis spectrophotometer, and the CIN concentration was determined based on a pre-established calibration curve. The release rate (R, %) was calculated according to Equation (5):

(5) $$R\;(\%)\;=\;\frac{M_{0}-M_{t}}{M_{0}}\;\times \;100$$

where *M*_0_ denotes the initial CIN loading in the film (mg), and *M_t_* represents the residual CIN extracted from the film at time t (mg).

### 2.7. Antimicrobial Properties

#### 2.7.1. Testing of Antibacterial Properties for Different Samples

Antibacterial performance testing against *E. coli* and *S. aureus* was conducted. Initial bacterial suspensions were obtained by growing each strain in nutrient broth at 37 °C with orbital shaking (120 rpm) for 24 h. For evaluation, film samples (30 mg) were combined with 10 mL of these suspensions and incubated similarly. Bacterial growth was tracked dynamically by measuring OD600 at intervals over a 29 h span using UV-Vis spectroscopy [24]. In parallel, a static endpoint method was employed: after exposure, 100 µL of each mixture was plated on agar, incubated for 24 h under controlled conditions, and the antibacterial efficacy was quantified by comparing the number of colonies formed in test samples versus controls.

#### 2.7.2. Test of the Humidity-Responsive Antibacterial Performance

Prior to the antibacterial assay, the film samples were equilibrated at 25 °C under target RH conditions (43%, 75%, and 98% RH) for one week to ensure moisture sorption equilibrium. Thereafter, circular film disks measuring 8 mm in diameter were excised and positioned onto LB agar plates that had been pre-coated with 100 μL of bacterial suspension. The plates were subsequently cultured at 37 °C for a period of 16 h. Following incubation, the diameters of the zones of inhibition were assessed and documented using a vernier caliper.

### 2.8. Statistical Analysis

Each set of experiments was carried out three times, and the outcomes were presented as the mean ± standard deviation (SD). Employ SPSS 27 software to conduct a one-way analysis of variance (ANOVA) followed by Tukey’s HSD post hoc test to identify significant differences between the groups, with a significance threshold set at *p* < 0.05. Graphs of the experimental data were generated using Origin Pro 2021.

## 3. Results and Discussion

### 3.1. Characterization of CIN@β-CD ICs

**Figure 1:**
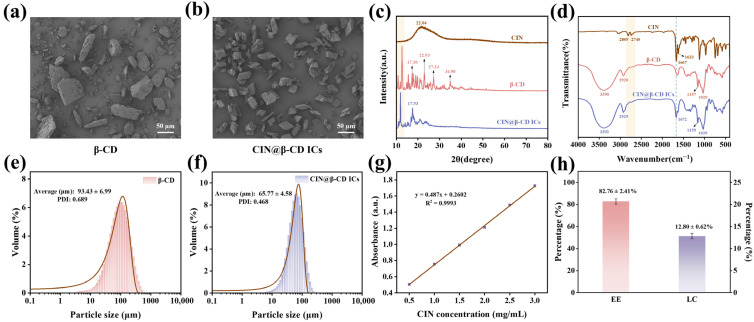
Characterization of CIN@β-CD ICs. (**a**,**b**) SEM image of β-CD and ICs, (**c**) XRD pattern, (**d**) FT-IR spectra, (**e**,**f**) particle size of β-CD and ICs, (**g**) standard fit curve of CIN, and (**h**) EE and LC of CIN@β-CD ICs.

As shown in Figure 1a,b, the pure β-CD exhibits an irregular block-like morphology, whereas the CIN@β-CD ICs display a regular, sharply edged prismatic structure. This significant morphological transformation is attributed to the entry of CIN into the hydrophobic cavity of β-CD and the packing mode and crystallization behavior of β-CD molecules were changed [28]. XRD analysis further supports this observation (Figure 1c). The XRD pattern of β-CD shows intense peaks at 2θ = 10.80°, 12.63°, 17.30°, 22.93°, 27.24°, and 34.90°, consistent with its cage-type crystalline packing [29]. In contrast, the diffraction pattern of CIN@β-CD ICs shows notable alterations: the amorphous diffuse peak of CIN (2θ = 22.04°) disappears, and several characteristic peaks of β-CD (10.80°, 27.24°, and 34.90°) vanish, while the remaining peak positions shift and weaken in intensity. These results collectively confirm that CIN molecules have been effectively encapsulated into the cavity of β-CD, leading to the reorganization of the host molecule’s crystal structure. This finding aligns with the results reported by Tian et al. [30].

FT-IR spectral analysis was utilized to investigate the complex formation between β-CD and CIN (Figure 1d). The spectrum of pure CIN displays characteristic peaks at 2809 cm^−1^ and 2740 cm^−1^, corresponding to the C-H stretching vibrations of the aldehyde group. Additional peaks at 1667 cm^−1^ and 1623 cm^−1^ are assigned to the C=O stretching vibration and the aromatic C=C stretching vibration, respectively [31]. For β-CD, characteristic absorption bands are observed at 3390 cm^−1^ (O-H stretching), 2920 cm^−1^ (C-H asymmetric stretching of -CH_3_ and -CH_2_ groups), 1157 cm^−1^ (C-O-C stretching), and 1029 cm^−1^ (C-O stretching) [32]. The FT-IR spectrum of the CIN@β-CD ICs largely retains the characteristic bands of β-CD, showing peaks attributable to each component, albeit with variations in intensity. Notably, the CIN peak at 1667 cm^−1^ remains detectable in the ICs spectrum, although slightly shifted and diminished in intensity, confirming the successful incorporation of CIN into the β-CD cavity [20].

Figure 1e,f further verify the formation of CIN@β-CD ICs through particle size distribution analysis. Compared with β-CD (93.43 ± 6.99 μm), the CIN@β-CD ICs exhibited a smaller mean particle size (65.77 ± 4.58 μm) and a narrower size distribution (PDI: 0.468), which is ascribed to the host-guest interaction between CIN and β-CD that promotes the formation of uniform aggregates [33]. Based on the standard curve equation of CIN, the EE and LC of the ICs were calculated to be 82.76 ± 2.41% and 12.80 ± 0.62%, respectively, confirming the effective encapsulation of CIN within the β-CD cavity.

### 3.2. Micromorphology

**Figure 2:**
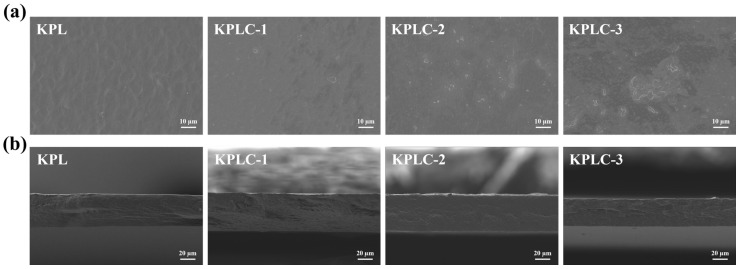
SEM micrographs of (**a**) Surface, (**b**) cross-section of KPL, KPLC-1, KPLC-2, and KPLC-3 films.

Composite films based on the KPL matrix with varying ICs content were fabricated. The surface and cross-sectional morphologies of the films were examined by SEM. Figure 2a shows that the KPL film exhibits a uniform and dense surface structure without obvious pores or defects, indicating the formation of a well-compatible network among KGM, PVA, and LiCl via hydrogen bonding interactions [34,35]. In contrast, the surfaces of the KPLC films show distinct morphological changes. Plate-like structures corresponding to the CIN@β-CD ICs are visible across all KPLC films, indicating that ICs incorporation introduced a multi-scale roughened surface. The KPLC-1 and KPLC-2 films show effective distribution of ICs within the matrix, without noticeable aggregation. Concurrently, the incorporation of ICs altered the discontinuous cross-section observed in the KPL film (Figure 2b), resulting in organized wrinkles and corrugations, which became more compact and potentially beneficial for enhancing mechanical properties [36]. However, at higher ICs content (KPLC-3), surface aggregation and a thinner cross-section were observed, suggesting potential overloading of ICs. A similar phenomenon has been reported for composite films loaded with pterostilbene@β-CD ICs [27].

### 3.3. FT-IR Analysis

**Figure 3:**
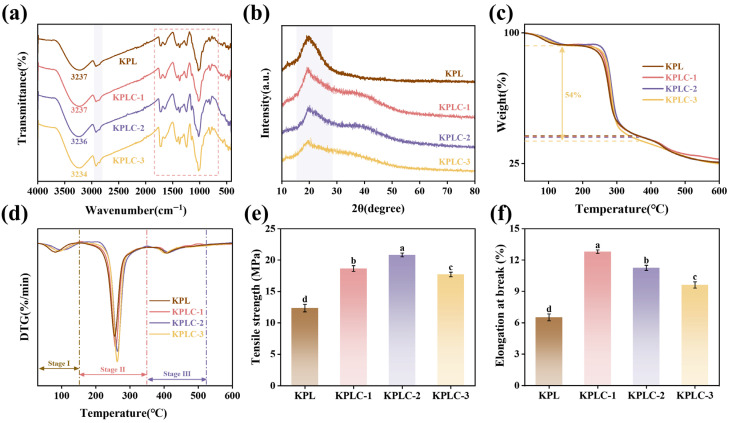
(**a**) FTIR spectra, (**b**) XRD pattern, (**c**) TGA, (**d**) DTG, (**e**) TS, and (**f**) EAB of KPL, KPLC-1, KPLC-2, and KPLC-3 films. Lowercase letters (a–d) indicate a significant difference (*p* < 0.05).

Figure 3a presents the FT-IR spectra of KPL and KPLC films. The broad absorption band for KPL film in the range of 3500–3000 cm^−1^ is assigned to O-H stretching vibrations; the absorption peak at 2930 cm^−1^ corresponds to C-H stretching vibrations; the absorption bands in the 1750–1500 cm^−1^ and 1020 cm^−1^ regions are attributable to C=O, C-O-C, and C-O stretching vibrations, respectively [37,38]. Upon incorporation of the CIN@β-CD ICs, the KPLC films retained most of the characteristic bands of the KPL matrix. Notably, the O-H absorption peak became narrower and shifted to a lower wavenumber, which is due to hydrogen bonding interactions between KPL and the ICs [25]. More pronounced variations in the peak shapes at 2930 cm^−1^ and 1715 cm^−1^ reflect that the ICs altered the local chemical environment within the KPL matrix, indirectly proving the successful introduction of ICs into the KPL film [31]. Furthermore, the intensity of the C-O absorption peak at 1020 cm^−1^ gradually increased with ICs content. This spectral modification confirms enhanced intermolecular interactions and the formation of a more compact hydrogen-bonded network within the composite films [24]. Importantly, these results show a concentration-dependent trend, implying an increase in the bonding strength of KPLC at higher ICs loading, which may underlie the improved film properties, although further experimental validation is still required.

### 3.4. XRD Analysis

The crystal structure and crystallinity of the films were analyzed using XRD (Figure 3b). The XRD pattern of KPL film displayed a broad peak at 2θ = 20.21°, confirming the predominantly amorphous nature of KGM, in agreement with earlier reports [39]. All KPLC films similarly exhibited a broad diffuse peak near 20.5°, indicating that their overall structure remained largely amorphous. Notably, with the addition of CIN@β-CD ICs, the patterns of KPLC-1, KPLC-2, and KPLC-3 films each showed an additional broadened peak in the range of 2θ = 36–40°. However, this diffraction peak was not observed in the XRD pattern of the ICs alone (Figure 1c). This phenomenon indicates that the aggregated state of the ICs within the composite film differs from that of the isolated ICs, likely due to hydrogen bonding interactions within the film [40]. The reorganization of the hydrogen-bonding network by Li^+^ ions further promoted the formation of such interactions [14]. With increasing ICs content, the intensity of the broad peak at around 20° gradually decreased. These changes can be ascribed to strong hydrogen bonding interactions between KPL and the CIN@β-CD ICs, suggesting a reduction in the overall crystalline order of the composite films [41]. Films with such structural characteristics generally allow faster moisture penetration due to less restricted diffusion of water molecules into the polymer network [42], a characteristic that could enhance the humidity-responsive behavior of the present composite films.

### 3.5. Thermal Stability

The thermal stability was evaluated by TGA and DTG curves. As shown in Figure 3c,d, all films underwent a similar three-stage weight loss process. The first stage primarily involved mass change due to the evaporation of bound water. The maximum weight loss occurred in the second stage, where the films thermally degraded between 150 °C and 360 °C, resulting in a mass loss of 52–54%. This loss is associated with glycerol decomposition and moisture loss caused by the breakage of intermolecular hydrogen bonds [36]. The third stage was attributed to carbonization following thermal degradation, with the final carbon residue percentages fluctuating within a relatively narrow range. The DTG curves further revealed that the initial exothermic peaks for the KPL, KPLC-1, KPLC-2, and KPLC-3 films appeared around 100 °C. This early peak is primarily due to KGM, a high-molecular-weight polysaccharide that retains more bound water and thus undergoes earlier thermal decomposition [43]. Furthermore, the maximum decomposition temperatures for the different films were 276.7 °C, 278.8 °C, 284.6 °C, and 283.7 °C, respectively. Compared with the original KPL film, the incorporation of an appropriate amount of CIN@β-CD ICs enhanced the thermal stability of the KPLC films. This improvement is primarily attributed to the hydrogen bonding and enhanced intermolecular association between the CIN@β-CD ICs and the KGM/PVA matrix [44]. However, excessive ICs led to uneven dispersion within the film matrix, which induced internal structural instability and consequently reduced thermal stability [45].

### 3.6. Mechanical Properties

TS and EAB were measured to evaluate the mechanical performance of the films. TS represents the maximum stress sustained before fracture, and EAB describes the material’s extensibility and flexibility [39]. Figure 3e shows that the TS of the KPL film was 12.35 ± 0.61 MPa. Increasing the content of CIN@β-CD ICs initially raised the TS of the KPLC films, with values reaching 18.65 ± 0.45 MPa (KPLC-1), 20.83 ± 0.32 MPa (KPLC-2), and 17.72 ± 0.37 MPa (KPLC-3), after which it declined at the highest loading. The highest TS observed for the KPLC-2 film is attributed to the relatively uniform distribution of ICs within the KPL matrix at this concentration, which strengthens intermolecular interactions and promotes a more cohesive network structure [46]. In contrast, the decrease in TS for KPLC-3 likely results from aggregation of excess ICs, which compromises film homogeneity and reduces mechanical integrity. Figure 3f indicates that the EAB value for the KPL film was 6.51 ± 2.15%. All KPLC films exhibited higher EAB than KPL, indicating that ICs incorporation improved film toughness. However, differing slightly from the trend in TS, the EAB gradually decreased as the amount of added ICs increased. Overall, the addition of ICs enhanced the tensile strength of the films while also modulating their toughness. This optimization of mechanical properties supports the potential use of these films in humidity-responsive actuating applications [14].

### 3.7. WCA, WVP, and MS Analysis

The films’ responsiveness to ambient moisture was evaluated using three complementary yet independent metrics: WCA, WVP, and MS (Table 1). Each parameter describes a distinct aspect of water-material interaction.

WCA quantifies the initial surface wettability. All KPL-based films showed contact angles below 90°, confirming their hydrophilic nature [47]. The WCA decreased in the order KPL > KPLC-1 > KPLC-2 > KPLC-3, indicating enhanced surface hydrophilicity. This can be attributed to the structure of β-CD, which has a hydrophilic outer surface and a hydrophobic cavity. This structure collectively promotes more rapid water spreading on the film [19]. The lower WCA implies a reduced energy barrier for water droplet spreading, which is conducive to the initial adsorption of environmental moisture—a prerequisite for triggering subsequent bulk interactions.

WVP reflects the film’s inherent capacity to transmit water vapor. The WVP gradually decreased with increasing CIN@β-CD ICs content, indicating improved passive moisture barrier performance in the KPLC films. Although the difference between KPLC-2 and KPLC-3 was not statistically significant, the overall reduction in WVP is likely due to hydrogen bonding between the ICs and the KPL matrix, which promotes the formation of a denser network structure [7,48]. This modified structure is critical as it dictates the kinetics of water vapor ingress, which is the primary driving force for activating the humidity-responsive mechanism in packaging applications.

MS measures the equilibrium water uptake capacity of the film. The MS rate of the films increased with rising environmental RH but decreased with ICs incorporation under the same RH. This reduction can be explained by the hydrophobic CIN molecules occupying the cavities of β-CD, decreasing the number of available hydration sites [49]. Notably, the KPLC films (especially KPLC-2) exhibited a pronounced contrast in MS between low-humidity (43% RH, 18.65%) and high-humidity (98% RH, 42.24%) conditions. This marked humidity-dependent swelling reflects a high structural sensitivity to moisture, establishing the essential bulk hydration condition for subsequent molecular events [26].

### 3.8. Humidity-Responsive Release Kinetics

**Figure 4:**
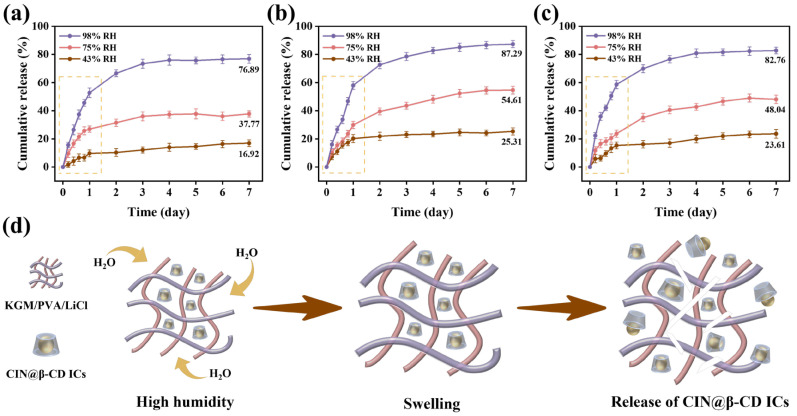
Humidity-responsive release of 43% RH (**a**), 75% RH (**b**), 98% RH (**c**), and (**d**) humidity-responsive release mechanism.

The cumulative CIN release from all films was consistently higher at 98% RH than at 75% or 43% RH (Figure 4a–c), demonstrating a pronounced humidity-dependent release behavior. Among the formulations, KPLC-2 exhibited the highest cumulative release. For instance, after 7 days at 98% RH, KPLC-2 released 87.29% of its loaded CIN, compared to 76.89% for KPLC-1 and 82.76% for KPLC-3. This result suggests that an optimal ICs loading exists, as a higher content does not linearly improve release performance [48].

To quantitatively elucidate the release mechanism, the data were fitted with various kinetic models (Figures S1–S3, Table 2). The Korsmeyer-Peppas model yielded release exponents (*n*) below 0.45 for all films under all humidity conditions, confirming a Fickian diffusion-dominated release process. Concurrently, the first-order model provided the best fit (highest R^2^ > 0.98 in most cases), reinforcing that the release rate was proportional to the remaining CIN concentration within the film matrix [28].

Further analysis of the release curves revealed a characteristic two-stage release pattern for CIN from the films under different RH conditions. The initial 24 h constituted a rapid release stage, quantified as an initial burst release of 57.98% for KPLC-2 at 98% RH. This stage is attributed to the rapid swelling of the hydrophilic matrix upon water infiltration, which instantly creates diffusion pathways for ICs near the surface [13,50]. Subsequently, the release entered a sustained phase. The time required to reach 90% of the final release amount (T_90_) was approximately 1.4 days for KPLC-2 at 98% RH, indicating an efficient transition to a prolonged, slow-release plateau. This sustained stage is governed by the slower diffusion of CIN from more deeply embedded ICs, a process modulated by the β-CD cavities and the polymer network [51].

Notably, the kinetic parameters quantitatively distinguished the formulations. KPLC-1 shows a relatively higher *n* value at low humidity, suggesting a potential initial rapid release tendency (e.g., 52.73% at 24 h, 98% RH); KPLC-2 demonstrated the most stable and predictable release kinetics, as evidenced by consistently high R^2^ values for both models across the tested humidity range; and the lower *n* value of KPLC-3 confirms the diffusion hindrance caused by excessive inclusion complex aggregation [52]. Overall, the rapid initial response coupled with sustained release makes KPLC films promising candidates for intelligent packaging of perishable foods.

### 3.9. Antimicrobial Activities

**Figure 5:**
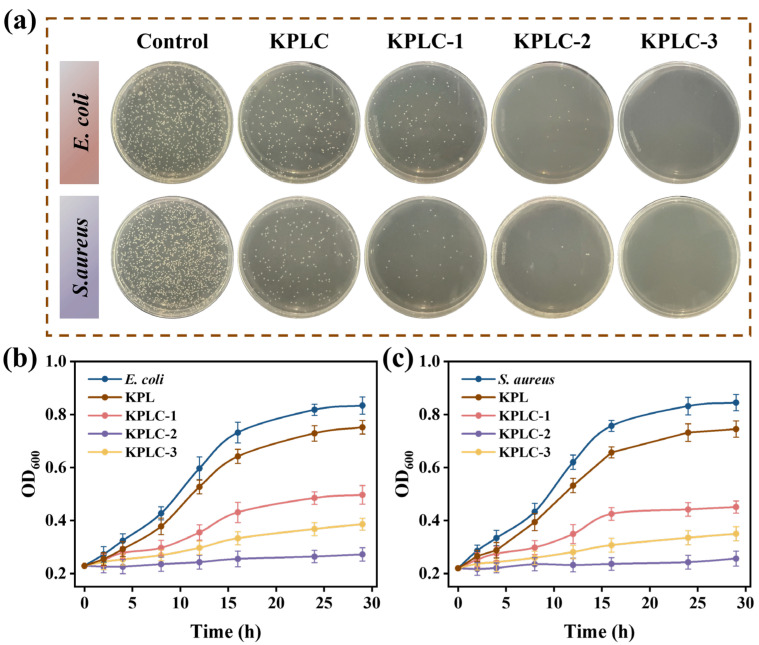
The antibacterial rate of *E. coli* and *S. aureus* treated with KPL, KPLC-1, KPLC-2, and KPLC-3 films (**a**). The antibacterial growth curves against *E. coli* (**b**), *S. aureus* (**c**).

The antibacterial activity of the films was preliminarily assessed through standard antibacterial rate tests. As shown in Figure 5a, the antibacterial rate of the films increased with higher CIN@β-CD ICs content, confirming that greater ICs loading enhances the initial release capacity of the antimicrobial agent. However, effective intelligent packaging requires sustained, long-term protection that correlates with the spoilage dynamics of food [6]. Therefore, microbial growth inhibition curves of the films were measured over a dynamic timeframe (Figure 5b,c). Interestingly, the dynamic growth curves showed that the KPLC-2 film (0.2% ICs) maintained the strongest antibacterial potency throughout the test cycle, which coincides with the optimal properties shown in the release kinetics (3.8 knots) and microstructure (3.2 knots). Although its initial antibacterial rate is not the highest, this just highlights the importance of the controlled release strategy for long-term protection: KPLC-1 may lead to insufficient subsequent release due to low ICs content; However, KPLC-3 may hinder the continuous release of active components due to the excessive aggregation of ICs (SEM observation). In contrast, the ICs of KPLC-2 are uniformly dispersed and the network structure is moderate, achieving an ideal balance between fast initial response and smooth and continuous release. Compared with similar films reported recently [19,44].

**Figure 6:**
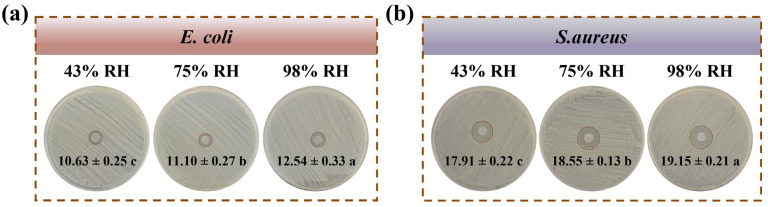
Inhibition zone against (**a**) *E. coli* and (**b**) *S. aureus* after storing for one week at room temperature, 43%, 75%, and 98% RH. Lowercase letters (a–c) indicate a significant difference (*p* < 0.05).

To assess the humidity-triggered release of CIN from the KPLC films and to confirm their intelligent responsiveness, the bacterial inhibition zone of the KPLC-2 film was measured under varying RH conditions. As shown in Figure 6, the inhibition zone diameter increased markedly with rising environmental humidity. This enhancement is ascribed to the greater expansion of the KPL polymer matrix upon hydration and more pronounced conformational changes in β-CD under high humidity, which together generate additional pathways for molecular transport within the film [49]. These structural changes accelerate the dissociation of CIN@β-CD ICs and promote the diffusion of CIN into the surrounding medium [20,53]. These observations align with previous results about the humidity-dependent CIN release and demonstrate that moisture-activated release directly enhances antibacterial efficacy.

## 4. Conclusions

In this study, a smart antimicrobial packaging film based on KGM/PVA/LiCl matrix and loaded with CIN@β-CD ICs was successfully developed. The results confirmed that CIN was efficiently encapsulated in β-CD (encapsulation rate: 82.76 ± 2.41%).The introduction of ICs improved the structural continuity of the matrix and formed a denser film network, which resulted in enhanced hydrophilicity, moisture sensitivity, and a reduction in water vapor permeability by about 26%. More importantly, the film exhibited excellent humidity-triggered antimicrobial activity, which could rapidly release CIN and effectively inhibit the growth of *E. coli* and *S. aureus* in high humidity environments (the diameter of the inhibitory circle was 12.54 ± 0.33 mm and 19.15 ± 0.21 mm, respectively).

The main advantage of this work is the construction of a synergistic, humidity-responsive release system through the effective integration of multi-component functional materials. However, there are some limitations to the study: although the LiCl enhancement of moisture sensitivity is consistent with the observed properties and previous literature, its specific contribution was not individually verified in this experimental system; moreover, the antimicrobial efficacy was only verified under controlled laboratory conditions, and the effectiveness of its application in real food systems still needs to be evaluated subsequently.

Therefore, future research could focus on the following: first, further increasing the CIN loading and evaluating its effect on the physical properties of the films; second, investigating the stability of the films under long-term storage or extreme environments; and finally, verifying the freshness preservation effect and safety in typical high-moisture perishable food products (e.g., freshly cut fruits and meats). This work provides a valuable theoretical reference and practical basis for the development of controlled release strategies using natural antimicrobial agents.

## Figures and Tables

**Table 1:** The WCA, WVP and MS of KPL, KPLC-1, KPLC-2, and KPLC-3 films.

Samples	WCA (°)	WVP (g mm/m^2^ h kPa)	MS (%)		
			43% RH	75% RH	98% RH
KPL	67.18 ± 0.35 ^a^	2.92 ± 0.57 ^a^	19.93 ± 0.47 ^a^	26.52 ± 0.43 ^a^	43.07 ± 0.23 ^a^
KPLC-1	54.41 ± 0.59 ^b^	2.34 ± 0.45 ^b^	19.58 ± 0.29 ^b^	25.58 ± 0.25 ^b^	42.89 ± 0.40 ^b^
KPLC-2	53.32 ± 0.43 ^c^	2.17 ± 0.31 ^c^	18.65 ± 0.33 ^c^	24.74 ± 0.27 ^c^	42.24 ± 0.36 ^c^
KPLC-3	51.65 ± 0.52 ^d^	2.16 ± 0.55 ^c^	17.97 ± 0.36 ^d^	23.02 ± 0.33 ^d^	41.22 ± 0.34 ^d^

Lowercase letters (a–d) indicate a significant difference (*p* < 0.05).

**Table 2:** Resulting parameters of CIN release in different RH with different models.

Samples	RH	Zero-Order (R^2^)	First-Order (R^2^)	Higuchi Model (R^2^)	Korsmeyer-Peppas (R^2^)	Korsmeyer-Peppas (n)
KPLC-1	43%	0.86189	0.95724	0.95931	0.96746	0.42825
	75%	0.65313	0.99012	0.82978	0.93423	0.26725
	98%	0.67624	0.99906	0.86578	0.92667	0.31842
KPLC-2	43%	0.55564	0.98977	0.79075	0.9263	0.24027
	75%	0.82175	0.98683	0.96347	0.97594	0.41075
	98%	0.70757	0.99437	0.89654	0.93431	0.35437
KPLC-3	43%	0.77394	0.93615	0.92739	0.95836	0.35081
	75%	0.82314	0.9758	0.96641	0.98254	0.39086
	98%	0.65167	0.99093	0.86943	0.95334	0.28561

## Data Availability

The original contributions presented in this study are included in the article/Supplementary Materials. Further inquiries can be directed to the corresponding authors.

## Supplementary Materials

The following supporting information can be downloaded at: https://www.mdpi.com/article/10.3390/foods15030464/s1. Figure S1. Modeled fitted curves for the release kinetics of CIN from KPLC-1 films under different RH environments: (A) zero-order model, (B) one-order model, (C) Higuchi model, and (D) Korsmeye–Peppas model. Figure S2. Modeled fitted curves for the release kinetics of CIN from KPLC-2 films under different RH environments: (A) zero-order model, (B) one-order model, (C) Higuchi model, and (D) Korsmeye–Peppas model. Figure S3. Modeled fitted curves for the release kinetics of CIN from KPLC-3 films under different RH environments: (A) zero-order model, (B) one-order model, (C) Higuchi model, and (D) Korsmeye–Peppas model. Figure S4. FTIR spectra of KPLC-2 films after different humidity treatments. Table S1. Calculated densities of KPL, KPLC-1, KPLC-2, and KPLC-3 films. Table S2. Thickness of KPL, KPLC-1, KPLC-2, and KPLC-3 films. Table S3. MIC and MBC of CIN@β-CD ICs against *S. aureus* and *E. coli*. References [24,32,54] are cited in the supplementary materials.
