# Supplementary material for: A Konjac Glucomannan-Based Antibacterial Packaging Film with Humidity-Triggered Release of Cinnamaldehyde
Source: Foods. 2026 Jan 29;15(3):464. doi: 10.3390/foods15030464 (PMC12896391; doi:10.3390/foods15030464)
Supplement: Supplementary file 1 [file foods-15-00464-s001.zip › foods-4079673-supplementary.pdf]

A konjac glucomannan-based antibacterial packaging film with humidity-triggered release of cinnamaldehyde

Yibin Chen [1], Hao Liu [3], Kaijun Sun [1], Qibiao Weng [4], Ying Yan [1], Liping Xiao [1], Ziwei Ye [1], Chengrong Wen [1], Jie Pang [1,*], Qian Ning [2,*]

[1] College of Food Science, Fujian Agriculture and Forestry University, Fuzhou 350002, China
[2] Jinshan College of Fujian Agriculture and Forestry University, Fuzhou 350002, China
[3] Fujian Dawuyi Green Food Research and Development Center, Nanping 353000, China
[4] Fujian Provincial Key Laboratory of Eel Breedingand Processing, Fuzhou 350003, China
* Correspondence: pang3721941@163.com (J. Pang), qianqian1000@163.com (Q. Ning)

Supplementary Methods:

Method S1. Cross-Linking Density

The cross-linking density of the films was determined through equilibrium swelling experiments based on the Flory-Rehner theory [1]. Briefly, pre-weighed dry film samples were immersed in deionized water at room temperature until swelling equilibrium was reached (no further weight change). The weight of the swollen samples was recorded after carefully removing surface moisture. The polymer volume fraction in the swollen film (v_{2m}) was calculated from the initial dry volume and the volume of absorbed water. The effective cross-linking density (v), defined as the number of network chains per unit volume, was then calculated using the Flory-Rehner equation.

$$v_{2m} = \frac{V_p}{V_\delta} = \frac{V_\delta - V_\omega}{V_\delta} = 1 - \frac{m_\omega \rho_\delta}{\rho_\omega m_\delta}$$

ρ_δ is the density of the swollen polymer, ρ_ω is the density of water, m_δ is the mass of the swollen polymer at equilibrium and m_ω is the mass of water present in the swollen polymer.

Supplementary Figures:

Table S1. Calculated densities of KPL, KPLC-1, KPLC-2, and KPLC-3 films.

Samples	Density (g/cm^3)
KPL	1.01 ± 0.09
KPLC-1	1.25 ± 0.14
KPLC-2	1.34 ± 0.27
KPLC-3	1.42 ± 0.15

Table S2. Thickness of KPL, KPLC-1, KPLC-2, and KPLC-3 films.

Samples	Thickness(μm)
KPL	36.40 ± 0.42
KPLC-1	49.61 ± 0.28
KPLC-2	52.35 ± 0.17
KPLC-3	54.12 ± 0.14

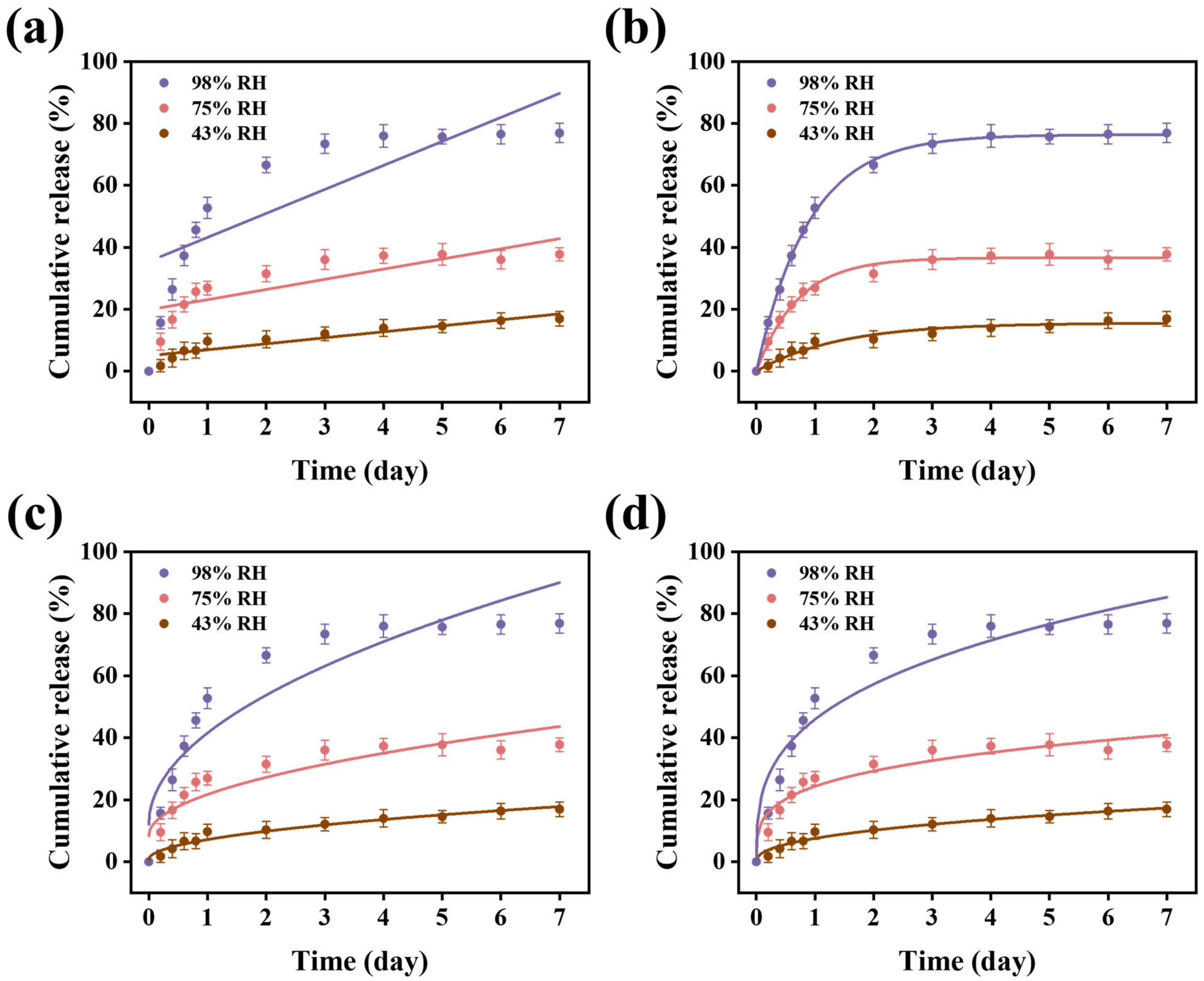

Figure S1. Modeled fitted curves for the release kinetics of CIN from KPLC-1 films under different RH environments: (**A**) zero-order model, (**B**) one-order model, (**C**) Higuchi model, and (**D**) Korsmeye–Peppas model.

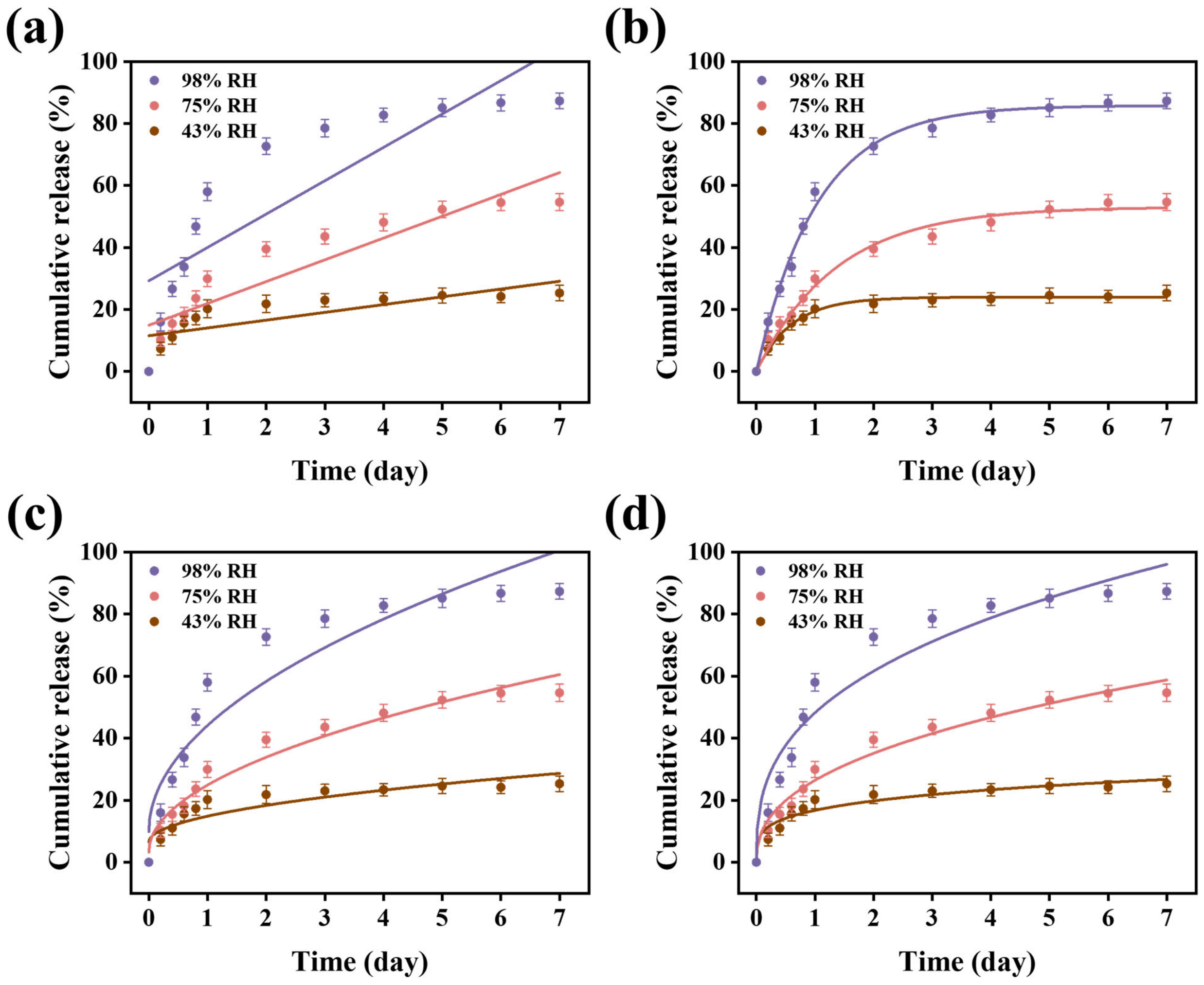

Figure S2. Modeled fitted curves for the release kinetics of CIN from KPLC-2 films under different RH environments: (**A**) zero-order model, (**B**) one-order model, (**C**) Higuchi model, and (**D**) Korsmeye–Peppas model.

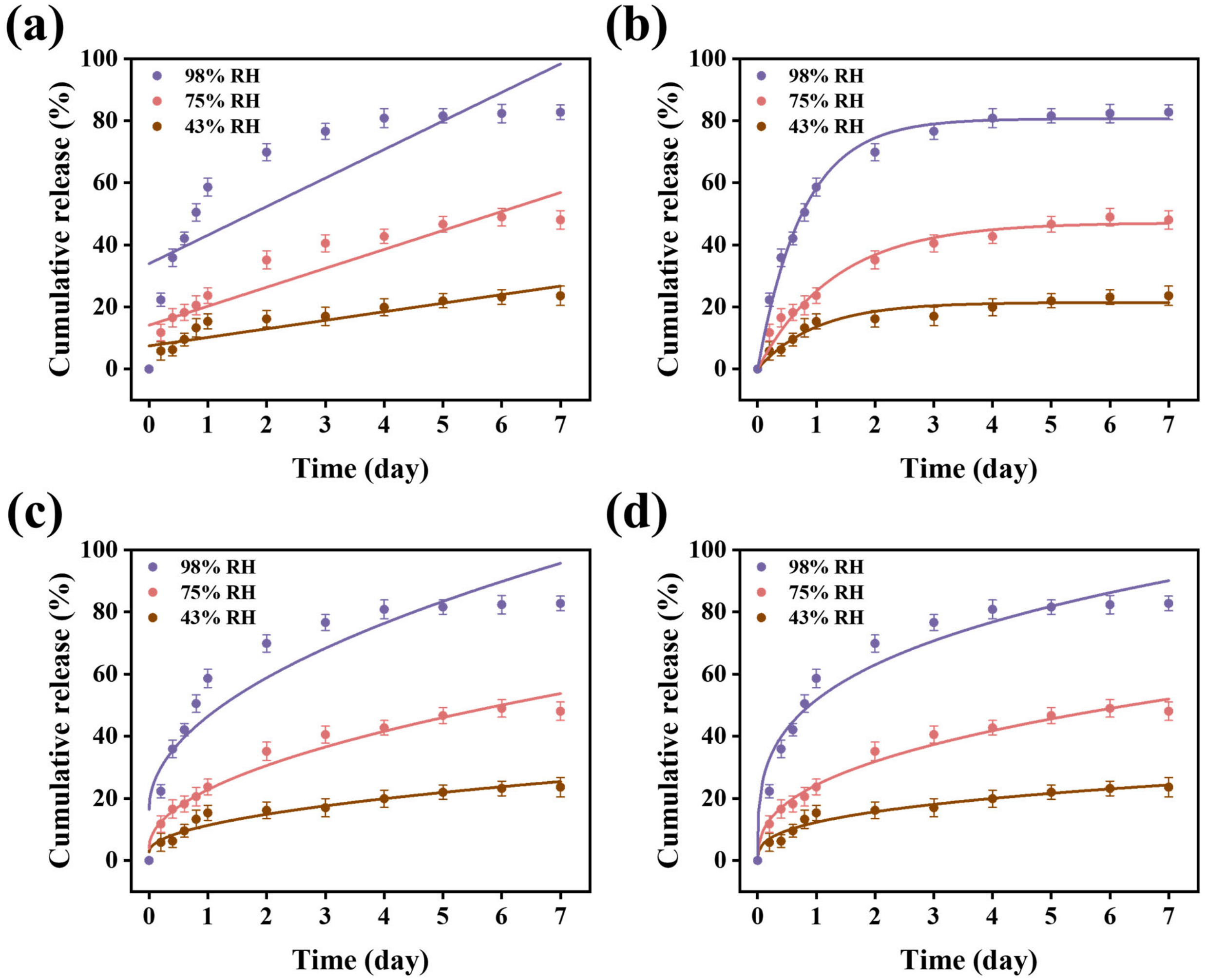

Figure S3. Modeled fitted curves for the release kinetics of CIN from KPLC-3 films under different RH environments: (**A**) zero-order model, (**B**) one-order model, (**C**) Higuchi model, and (**D**) Korsmeye–Peppas model.

FTIR spectra (**Figure S4**) provided insights into the molecular-level interactions of β-CD within the KPLC-2 films. The shift in the O-H band to a lower wavenumber (from 3454 cm^{-1} to 3450 cm^{-1}) demonstrates the reorganization of the hydrogen-bonding network [2]. Importantly, the relative intensity of β-CD's characteristic peaks (e.g., 1157 and 1029 cm^{-1}) changed significantly after humidity treatment. In FTIR spectroscopy, such intensity variations are sensitive indicators of altered local constraints and vibrational coupling around the β-CD molecules [3]. Collectively, these spectral changes confirm that β-CD experiences a dynamically reconfigured microenvironment.

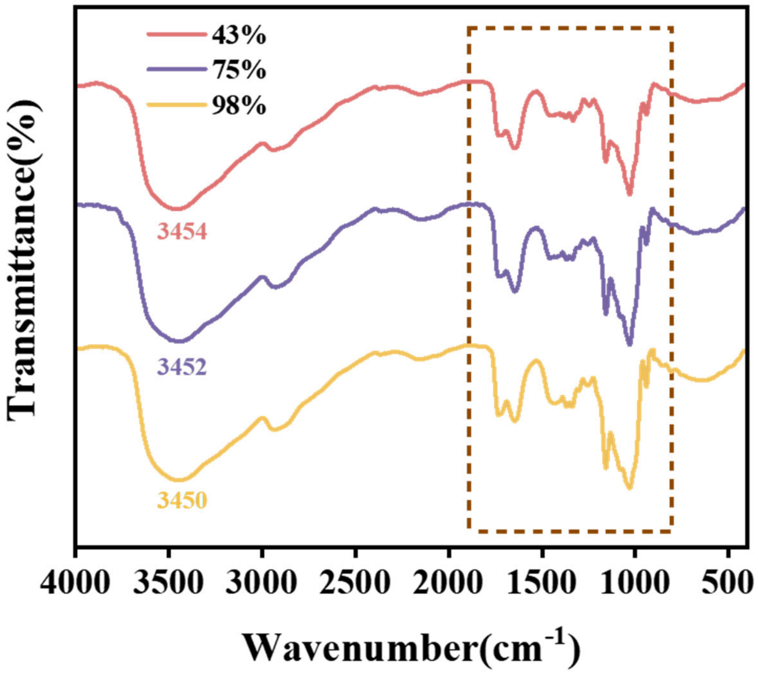

Figure S4. FTIR spectra of KPLC-2 films after different humidity treatments.

Table S3. MIC and MBC of CIN@β-CD ICs against *S. aureus* and *E. coli*.

	MIC (mg/mL)	MBC (mg/mL)
S. aureus	1.25	3.75
E. coli	1.25	3.75

References

1. Hoti, G.; Caldera, F.; Cecone, C.; Rubin Pedrazzo, A.; Anceschi, A.; Appleton, S.L.; Khazaei Monfared, Y.; Trotta, F. Effect of the Cross-Linking Density on the Swelling and Rheological Behavior of Ester-Bridged β-Cyclodextrin Nanosponges. *Materials* **2021**, *14*, 478, doi:10.3390/ma14030478.
2. Wu, Z.; Wang, L.; Hu, Z.; Guan, X.; Chen, Y.; Xu, M.; Chen, X.; Bu, N.; Duan, J.; Liu, W.; et al. Konjac Glucomannan/Zein Active Film Loaded with Tea Polyphenol–Ferric Nanoparticles for Strawberry Preservation. *International Journal of Biological Macromolecules* **2025**, *299*, 139905, doi:10.1016/j.ijbiomac.2025.139905.
3. Han, Y.; Jia, F.; Bai, S.; Xiao, Y.; Meng, X.; Jiang, L. Effect of Operating Conditions on Size of Catechin/β-Cyclodextrin Nanoparticles Prepared by Nanoprecipitation and Characterization of Their Physicochemical Properties. *LWT* **2022**, *153*, 112447, doi:10.1016/j.lwt.2021.112447.
